# Extracellular vesicles microRNA-592 of melanoma stem cells promotes metastasis through activation of MAPK/ERK signaling pathway by targeting PTPN7 in non-stemness melanoma cells

**DOI:** 10.1038/s41420-022-01221-z

**Published:** 2022-10-27

**Authors:** Yuhan Zhang, Yan Chen, Lei Shi, Jie Li, Wenjuan Wan, Bowen Li, Doudou Liu, Xiaoshuang Li, Yuting Chen, Meng Xiang, Hao Chen, Bin Zeng, H. Rosie Xing, Jianyu Wang

**Affiliations:** 1 State Key Laboratory of Ultrasound in Medicine and Engineering, College of Biomedical Engineering, Chongqing Medical University, 400016 Chongqing, China; 2 Chongqing Key Laboratory of Biomedical Engineering, Chongqing Medical University, 400016 Chongqing, China; 3 Institute of Life Sciences, Chongqing Medical University, Chongqing, China; 4 Department of Ophthalmology, The First Affiliated Hospital of Chongqing Medical University, Chongqing Key Laboratory of Ophthalmology and Chongqing Eye Institute, Chongqing, China; 5 Present Address: Chongqing Health Center for Women and Children, Chongqing Key Laboratory of Human Embryo Engineering, Chongqing, China; 6 Present Address: Chongqing Medical University, Yi Xue Yuan Road, Yuzhong District 400016 Chongqing, P.R. China

**Keywords:** Cancer microenvironment, Cancer stem cells

## Abstract

Melanoma, one of the most aggressive malignancies, its high mortality and low survival rates are associated with effective metastatic colonization. Melanoma metastasis hinges on the bidirectional cell-cell communication within the complex metastatic microenvironments (MME). Extracellular vesicles (EVs) are recognized as a new class of molecular mediator in MME programing. Published studies show that melanoma EVs can educate MME stromal cells to acquire the pro-metastatic phenotype to enhance metastatic colonization. Whether EVs can mediate the interactions between heterogenous cancer cells within the MME that alter the course of metastasis has not been investigated at the mechanistic level. In this study, melanoma parental cells (MPCs) and paired derivative cancer stem cell line melanoma stem cells (MSCs) that were derived from melanoma cell line M14 were used. We demonstrate that the EVs-mediated crosstalk between the MSCs and the MPCs is a novel mechanism for melanoma metastasis. We characterized miR-592, a relatively novel microRNA of prognostic potential, in mediation of such intercellular crosstalk. EVs can encapsulate and deliver miR-592 to target MPCs. Upon entering, miR-592 inhibits the expression of its gene target protein tyrosine phosphatase non-receptor type7 (PTPN7), a phosphatase targeting MAPKs. This leads to the relief of the inhibitory effect of PTPN7 on MAPK/ERK signaling and consequently the augmentation of metastatic colonization of MPCs. Thus, via the extracellular vesicle miR-592/PTPN7/MAPK axis, melanoma-CSCs can transfer their metastatic ability to the low-metastatic non-CSC melanoma cells.

## Introduction

Melanoma has become one of the fastest growing malignancies in the world. Mortality of metastatic melanomas is on the rise whereas the cure rate is less than 10% [1]. The low survival rate of melanoma patients is due to effective metastatic colonization [2]. Conventional therapies, as well as immunotherapy and personalized therapies, have shown limited efficacy for metastatic melanoma [3–5]. Metastatic progression and development of treatment resistance are the bottleneck for melanoma treatment. Thus, there is a deep need to investigate the complexity of melanoma metastasis at the mechanistic level.

Cancer stem cells (CSCs)—a small subpopulation of cancer cells, have been proposed as cancer-initiating cells. CSCs are characterized by self-renewal properties and are involved in all aspects of cancer [6, 7]. Involvement of CSCs in melanoma has been reported, such as in epithelial-to-mesenchymal transition (EMT) and angiogenesis, as well as in metabolic reprogramming [8, 9]. CSCs in melanoma are low immunogenic and have the ability to evade immune surveillance [10]. CSCs also contributes to the heterogeneity of melanomas.

EVs are small extracellular vesicles that contain genetic material, proteins and lipids. EVs mediate the crosstalk between tumor cells and the stromal cells in the tumor microenvironment (TME) [11, 12]. Tumor-derived EVs are emerging mediators of tumorigenesis. Elucidating the role of EVs in facilitating the interactions between cancer cells and the MME has been focused on cancer cell-derived EVs in modulating the functions of stromal cells in the MME. EVs secreted by cancer cells can alter the TME and promote the pre-metastatic niche formation and metastasis [13]. Tumor-released EVs-miRNAs are associated with TME reprogramming. MicroRNAs are small non-coding RNAs with the ability of regulating gene expression at the post-transcriptional level either by inhibiting mRNA translation or by promoting mRNA degradation [14]. The involvement of miRNAs in cancer development and progression has been extensively investigated [14]. In melanoma, miR-1908, miR-199a-5p, and miR-199a-3p can target apolipoprotein E (ApoE) synergistically to augment metastatic invasion and colonization [15]. Our previous studies have shown that EVs-miRNAs secreted by highly metastatic melanoma cells promote distant metastasis and colonization of low-metastatic melanoma cells [16, 17]. Increasing evidence indicates that CSCs can effectively utilize the multidirectional communication system established by EVs to achieve efficient distant metastasis. However, studies on CSCs regulation of melanoma progression via EVs-mediated intercellular crosstalks are very limited.

In this study, melanoma parental cells (MPCs) and paired derivative cancer stem cell line melanoma stem cells (MSCs) that were derived from melanoma cell line M14 were used [18]. We demonstrate that the EVs-mediated crosstalk between the MSCs and the MPCs is a novel mechanism for melanoma metastasis. Bioinformatics analysis of EVs-miRNA sequencing data has prioritized clinically significant “EVs miR-592-PTPN7-MAPK/ERK axis” for experimental validation. At the mechanistic level, our study characterized miR-592, a relatively novel microRNA of prognostic potential, in mediation of such intercellular crosstalk. Prior to this study, the oncogenic activity of miR-592 has only been reported in prostate cancer [19] and thyroid cancer [20]. MSCs can deliver their EVs miR-592 to the targeted MPCs. Upon entering MPCs, miR-592 inhibits the expression of its gene target protein tyrosine phosphatase non-receptor type7 (PTPN7), a phosphatase targeting MAPKs. This leads to the relief of the inhibitory effect of PTPN7 on MAPK/ERK signaling and consequently the augmentation of metastatic colonization of MPCs. Thus, via the extracellular vesicle miR-592/PTPN7/MAPK axis, melanoma stem cells (MSCs) can transfer their metastatic ability to the low-metastatic non-CSC melanoma cells. Further, we show the prognostic values of miR-592 and PTPN7 in melanoma.

## Method

### Cell Lines

Melanoma cell line M14 was provided by Dr. Robert Hoffman (University of California San Diego). As we previously reported, low-metastatic-OL cells were established by conducting three-rounds of in vivo passage, isolation and purification of the pulmonary metastatic foci. Only a limited number of metastases were produced on the mouse lungs after orthotopic injection of OL cells [17]. In this study, we name OL cells as melanoma parental cell (MPCs). Five consecutive rounds of single-cell cloning using melanoma parental cells (MPCs) were performed to establish the melanoma stem cell (MSCs) line as we described [18, 21] (Supplementary Fig. 1b). MPCs were cultured in DMEM supplemented with 10% fetal bovine serum (FBS) and 1% penicillin-streptomycin. MSCs were cultured in DME/F12 supplemented with 2% B27 and 1% penicillin-streptomycin. All cell culture products are acquired from Gibco.

### EVs isolation and characterization

EVs were extracted by ultracentrifugation. Twenty-four hours before supernatant collection, serum was removed and cells were cultured in serum-free medium. Supernatant was collected when cell growth reached about 90%. Supernatants were subjected to differential centrifugation and carried out at 4 °C following the order: at 800 × *g* for 10 min; at 10,000 × *g* for 45 min; and at 100,000 × *g* for 80 min. After ultracentrifugation, the supernatant was discarded and the pellet was resuspended in PBS and stored at 4 °C. EVs protein content was determined by microBCA assay (CWBIO, CW2011S). The lipid bilayer structure was examined by transmission electron microscopy (TEM, jem-1400plus). EVs markers were detected by Western blot. The particle size distribution and concentration (1 × 10^6^–5 × 10^6^) were measured by Nanoparticle Tracking Analysis (NCS300).

### Western Blot

100ul RIPA (Solarbio, R10010) was added to 1 × 10^6^ cells for lysis. Cells were placed on ice for 10 min and centrifuged at 12,000 × *g* for 20 min. 5× loading buffer (Solarbio, P1040) was added to the pellet and boiled for 10 min. Thirty to fifty micrograms protein/sample was loaded, separated by electrophoresis with 10–12% polyacrylamide gel, and transferred to a polypropylene fluoride membrane (PVDF; Solarbio, ISEQ00010). 200 ug EVs were frozen and thawed repeatedly and sonicated (KQ5200DE) for 30 min. Extraction of proteins from EVs is similar to that of the cells. 5× loading buffer was added to the pellet and boiled for 10 min. Antibodies were purchased from Proteintech, China. (Supplementary Table 2). Results were analyzed by ImageJ version 1.47 (National Institutes of Health, Maryland, USA). Three independent experiments were conducted for statistical analysis.

### Real-time quantitative PCR

Total cellular (1 × 10^6^) or EVs (200 ug) RNAs was extracted by RNA easy isolation reagent (Vazemy, R70101) and miRNeasy serum / plasma kit (Qiagen, 217184), respectively. Reverse transcription was performed by PrimeScriptrt main mixture kit (Takara, RR036A) and mir-x™miRNA first strand synthesis kit (Takara, 638315). Chamq universal SYBR qPCR Master Mix (Vazyme, Q311-02) was used for RT-qPCR reaction. The following PCR condition was used: pre-incubation at 95 °C for 30 s, followed by 39 cycles at 95 °C for 5 s and 60 °C for 30 s, respectively. The relative expression was analyzed by the 2 − ΔΔCt method. Refer to supplementary materials for detailed primer sequences (Supplementary table 1).

### Transwell migration and invasion assays

Invasion assay with Matrigel (30 μl, 1:8 dilution in serum-free medium, BD Biosciences) and migration assay without Matrigel were performed. Transwell inserts (8 μm pore size, BD Falcon) were used for both assays. 20,000–40,000 cells were added to the upper chamber that contained 300 μl serum-free medium. Seven hundred microliters DMEM (Gibco) with 20% serum (Gibco) was added to the lower chamber. After 22 h of incubation, the chamber was taken out and fixed with glacial methanol at 4 °C for 30 min, and then stained with crystal violet for 15 min. Four random fields from each membrane and triplicates Transwells were photographed. The number of migrated or invaded cells was presented as the number of cells counted per field of the porous membrane (Image J 1.47).

### CCK-8 assays

The proliferation ability was detected by CCK-8 cell counting kit (Solarbio, CA1210). 3000 cells were added to each well of the 96-well plate. 10ul CCK-8 reagent was added and incubated at 37 °C for 1.5 h. The optical density (OD) at 450 nm was measured after culture for 0, 24, 48, and 72 h, respectively. Each experiment was conducted in triplicates.

### Colony formation assays

Three hundred to one thousand cells were added to each well of a six-well plate. After 7–12 days of culture, cells were fixed with ice methanol for 30 min and stained with crystal violet for 10 min. The plate was imaged and colonies with more than 50 cells were counted. For colony formation assays involving the addition of PD98059 (MCE, HY-12028), PD98059 was used at a final concentration of 20 μM and added to the culture, the day after cell seeding. After 72 h of treatment with PD98059, cells were washed with PBS and replaced with normal medium.

### Animal experiments

Nod-SCID mice (6–8 weeks, male) were obtained from the animal experiment center of Chongqing Medical University. 5 × 10^5^cells/mouse were injected into the caudal vein, and the weight changes were recorded every day. Animals were randomly assigned. When weight drop was observed for 5 consecutive days, mice were sacrificed and the autopsy was performed. The heart, liver, spleen, lung and kidney were examined and dissected. The extent of metastasis was observed with sellstromz87 fluorescent goggles (LUYOR, 3430-RB, Shanghai). Samples from animals with the number of metastases <1 were excluded. Animal experiments were approved by Chongqing Medical University ethical committee (#SCXK2012-0001).

### Luciferase assay

PTPN7 wild and mutant plasmids were constructed by the company (Hanbio, Shanghai; Supplementary table 3). HEK-293T cells, seeded in the 96-well plate, were co-transfected with PTPN7 plasmid and miR-592 mimics for 48 h. For the luciferase assay, the following working solutions were prepared: A: 10 μl (DMEM) + 0.8 μl (plasmid) + 0.25 μl (mimics/NC); B: 10 μl (DMEM) + 0.24 μl (lip2000). After mixing Solution A and Solution B, let the mixture stand for 10 min, then added it to each well and incubated for 48 h. Fluorescence expression was detected using a kit (Dual-Luciferase Reporter Assay Kit, Hanbio) under a chemiluminescent microplate reader (Thermo, VARIOSKANLUX).

### Detection of Cy5-labeled EVs miR-592 transfer

miR-592 mimics-cy5 (Genepharma, green) was transfected into MSCs. Supernatant was collected and EVs extracted as describing above. Two hundred micrograms EVs were labeled with PKH26 according to the instructions in the product information sheet (Umibio, UR52302). PKH26-labeled EVs were co-cultured with 1 × 10^5^ MPC cells. Cells were collected at 0, 6, 9, and 12 h, respectively. After washing with PBS, cells were fixed with ice-cold methanol for 10 min. Cell nuclei were stained with DAPI (Solarbio, C0060). All images were taken by confocal fluorescence microscopy (ANDOR, Dragonfly200).

### Statistical analyses

All experiments were repeated to obtain three independent biological replicates. The quantitative results were shown as mean ± SEM (standard error of mean). Statistical charts were supplied by GraphPad Prism8.0.2 draw (ns, not significant; *P < 0.05; **P < 0.01; ***P < 0.001). Proliferation curves were drawn using graphpadprism9, and all statistical analysis was performed by two-tailed Student’s *t*-test. The numbers of migrated cells and invaded cells, as well as lung metastases were counted by Image J(win32).

## Results

### EVs secreted by melanoma stem cells can enhance the invasiveness of melanoma parental cells in vitro

**Fig. 1:**
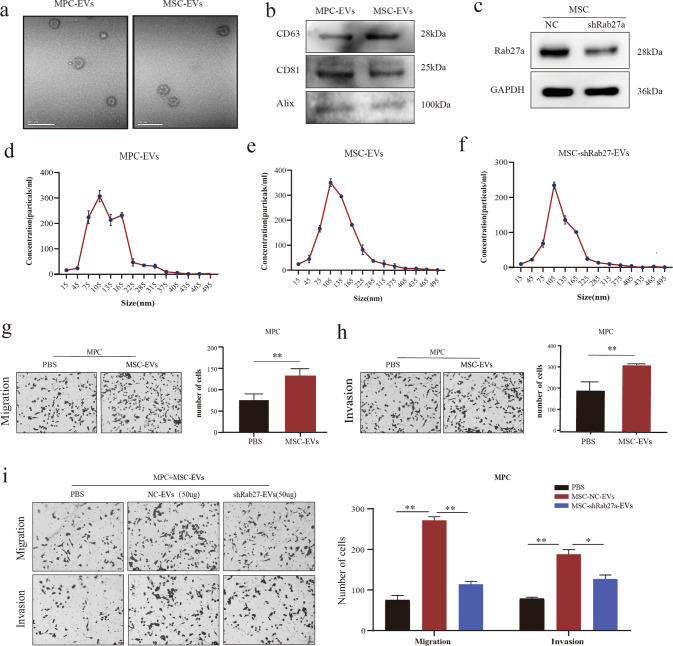
EVs from MSCs enhance the invasiveness of non-CSC MPCs. **a** EVs isolated from MPCs and MSCs were observed by transmission electron microscopy. **b** EVs marker proteins were detected by western blot. **c** Rab27a knockdown efficiency. **d**–**f** EVs particle size and concentration (1 × 10^6^–5 × 10^6^) were identified by Nanoparticle Tracking Analysis. **g** MSC-EVs enhanced the migration ability of MPCs, *P* < 0.01. **h** MSC-EVs enhanced the invasion ability of MPCs, *P* < 0.01. **i** The effect of shRab27-MSC-EVs (50 ug, 24 h) on MPCs migration and invasion. (ns not significant; *P < 0.05; **P < 0.01; ***P < 0.001).

EVs were extracted and purified by ultracentrifugation (Methods 2.2). Typical lipid bilayer structure was observed under transmission electron microscope, showing that the average size of EVs was between 30 and 150 nm (Fig. 1a). The expression of EVs markers such as CD81, CD63, Alix (Fig. 1b) and the negative markers ALB and Calnexin (Supplementary Fig. 1a) were detected by Western blot. Nanoparticle tracking analysis showed that the peak particle size of EVs was around 100 nm (Fig. 1d, e).

It has been shown that melanoma EVs can educate bone marrow progenitor cells toward a pro-metastatic phenotype through MET [22]. Whether melanoma stem cells can enhance the aggressiveness of melanoma cells at the site of tumor metastasis has not been examined mechanistically. We investigated the effect of MSC-EVs on MPCs in vitro. Transwell analysis showed that the migration and invasion abilities of MPC cells were considerably enhanced after 50 ug MSC- EVs were added into MPCs (Fig. 1g, h). Rab27 family regulates EVs secretion. Thus, EVs secretion can be effectively inhibited by silencing Rab27a [23]. To confirm that the observed enhancement of MPCs invasiveness was the result of MSC-EVs treatment, we effectively silenced the expression of Rab27a in MSC, as confirmed by Real-time Quantitative PCR Detecting System(q-PCR), western Blot and NTA analyses collectively (Fig. 1c, f and Supplementary Fig. 2a). As anticipated, upon the silence of Rab27a in MSCs, compared with the NC, migration and invasion were both decreased in shRab27a-MSC-EVs (Fig. 1i). We also used the EVs inhibitor GW4869 for validation and the results were consistent (Supplementary Fig. 1g) These results indicate that MSCs can enhance the invasiveness of MPCs through EVs in vitro. Based on this set of observation, we hypothesized that high-metastatic melanoma stem cells (MSCs) can transfer their “metastatic ability” to the low-metastatic melanoma cells (MPCs) via the EVs route. Recent studies have demonstrated that EVs-miRNAs were involved in the remodeling and activation of the tumor microenvironment [14]. Therefore, in this study, we chose to investigate the mechanism underlying the EVs-miRNA pathway in mediation the interactions between CSC melanoma cells and non-CSC melanoma cells.

### EVs miR-592 from MSCs promotes the metastatic colonization ability of MPCs in vitro and in vivo

**Fig. 2:**
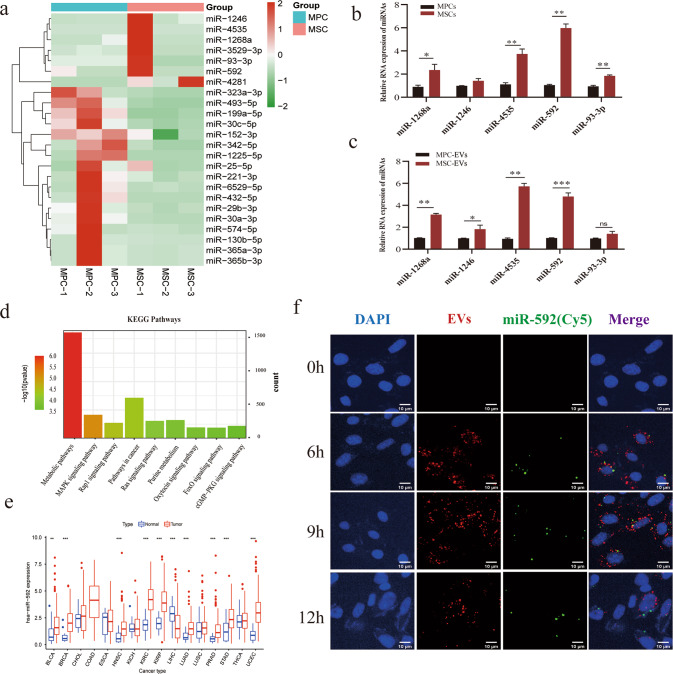
miR-592 is significantly overexpressed in MSC-EVs, can be encapsulated by MSC-EVs and transported to MPC cells. **a** The EVs sequencing results are shown by heatmap. **b** The expression of miR-592 in MSCs was higher than that in MPCs. **c** The expression of miR-592 in MSC-EVs was higher than that in MPC-EVs. **d** KEGG enrichment analysis for differentially expressed miRNAs. **e** Pan cancer analysis of miR-592 by TCGA database. **f** miR-592 (cy5, green) can be encapsulated by EVs (PKH26, red) and transported from MSCs to MPCs. (ns not significant; *P < 0.05; **P < 0.01; ***P < 0.001).

Based on EVs sequencing (BGI, Supplementary Methods 2.1), we found that seven microRNAs exhibited significantly higher expression in the EVs of MSCs, as visualized by R language (4.0.4) (Fig. 2a; Supplementary Fig. 1c). Five differentially expressed miRNAs (log2(FC) > 2 and *p* < 0.01) were chosen for further biological verification. RCR analysis showed that the expression of miR-1268a, miR-93-3p, miR-592, miR-4535 in MSCs were noticeably higher than those in MPCs (Fig. 2b). As expected, the expression of these miRNAs in the EVs of MSCs (MSC- EVs) was also higher than that in the EVs of MPC (MPC- EVs) (Fig. 2c).

In order to comprehend the function of these differentially expressed miRNAs, we performed a set of bioinformatics analysis. KEGG pathway analysis showed that differentially expressed miRNAs were mainly involved in MAPK, Ras, Rap1 signaling pathways and metabolic pathways as well as other cancer-associated pathways (Fig. 2d). GO analysis showed that the differentially expressed miRNAs were enriched in various biological processes and molecular functions. Cellular component analysis showed that miRNAs were highly enriched in cells, cell parts, cytoplasm, organelles (Supplementary Fig. 1d–f). Among the five prioritized miRNAs, we chose miR-592 for further mechanistic investigation because the function of miR-592 is largely unknown. Prior to this study, the oncogenic activity of miR-592 is only reported in pancreatic and prostate cancer [19, 20]. There are no reports of the involvement of miR-592 in cancer progression via the EVs route. The clinical relevance of miR-592 was evaluated by bioinformatics pan-cancer analysis. For cancers of different tissue origin, the expression of miR-592 in tumor tissues was higher than that in normal tissues for the majority, suggesting that miR-592 may function as a potential biomarker (Fig. 2e; Supplementary table 4).

**Fig. 3:**
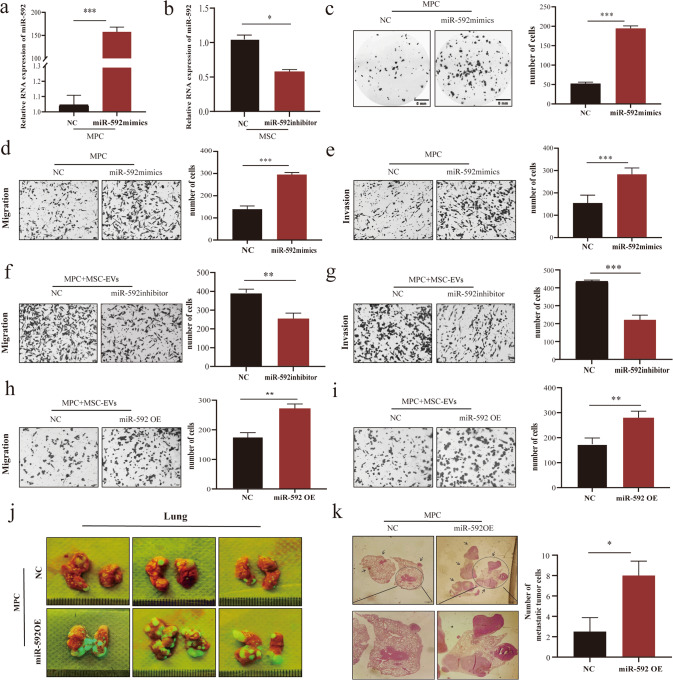
miR-592 promotes the invasiveness of MPC cells in vitro and in vivo. **a** Overexpression efficiency of miR-592 mimics treatment was confirmed in MPCs. **b** The knockdown efficiency of miR-592 inhibitor was confirmed in MSCs. **c**–**e** Effects of miR-592 overexpression on migration, invasion and colonization of MPCs in vitro. **f**, **g** Effects of miR592 inhibitor treatment on the migration and invasion of MPCs by Transwell assay in vitro. **h**, **i** Overexpressing miR-592 MSC-EVs enhanced the migration and invasion of MPCs in vitro. **j** Lung metastases were observed in mice receiving the tail vein injection of MPCs-miR-592-OE (green). **k** H&E staining observation and quantification of lung metastases. (ns not significant; *P < 0.05; **P < 0.01; ***P < 0.001).

The EVs tracing experiment confirmed that miR-592 can be encapsulated by EVs and enter MPCs (Fig. 2f; Methods 2.10). To verify whether miR-592 can affect the invasiveness of MPCs, we enhanced or inhibited miR-592 function by transfection of MPC cells with either miR-592 mimics or inhibitor, respectively. Changes in miR-592 expression in MPC cells and in EVs were confirmed by q-PCR, respectively (Fig. 3a, b and Supplementary Fig. 2b–e). While overexpression of miR-592 markedly enhanced the migration, invasion and colony formation ability of MPCs in vitro (Fig. 3c–e), miR-592 had no significant effect on the proliferation of MPC cells (Supplementary Fig. 2g). The above results suggests that miR-592 may primarily affect the metastatic characteristics of MPCs rather than proliferation. Inhibition of miR-592 activity in MSCs prevented the enhancement of migration and invasion of MPCs treated with MSC-EVs (Fig. 3f–g). In contrast, augmenting miR-592 activity in MSCs-EVs promoted the migration and invasion of MPCs (Fig. 3h–i). In order to more rigorously verify the function of miRNA-592 in melanoma, we also observed the same results in A375, another human malignant melanoma cell line (Supplementary Fig. 3c–h). This set of observations collectively indicates that the stimulative effect of MSC-EVs on MPC invasiveness in vitro requires the transfer of EVs miR-592 from MSC to MPCs.

We employed the mouse tail vein metastasis model to explore the effect of miR-592 on MPCs metastatic colonization ability in vivo (Methods Animal experiments). MPC cells stably overexpressing miR-592 (OE-miR592-MPC) were generated via lentiviral infection (Method, GeneChem, Shanghai). 6 × 10^5^/mouse NC-MPC cells and OE-miR592-MPC cells were injected into the tail vein of immunodeficiency Nod-Scid mice. When mice were sacrificed, gross autopsy was performed. More macroscopic metastases (*n* = 3) were observed in the lungs of mice receiving MPCs-miR-592-OE (Fig. 3j; Supplementary Fig. 3a, Supplementary Fig. 4a). H&E staining of lung biopsy confirmed that there were quantitatively and significantly more metastatic foci in mice receiving OE-miR592-MPC cells (Fig. 3k). Our observations demonstrate that miR-592 can enhance metastatic colonization efficiency of MPCs both in vitro and in vivo.

### PTPN7 mediates the pro-metastatic activity of EVs miR-592 via activation of the MAPK/ERK signaling pathway

**Fig. 4:**
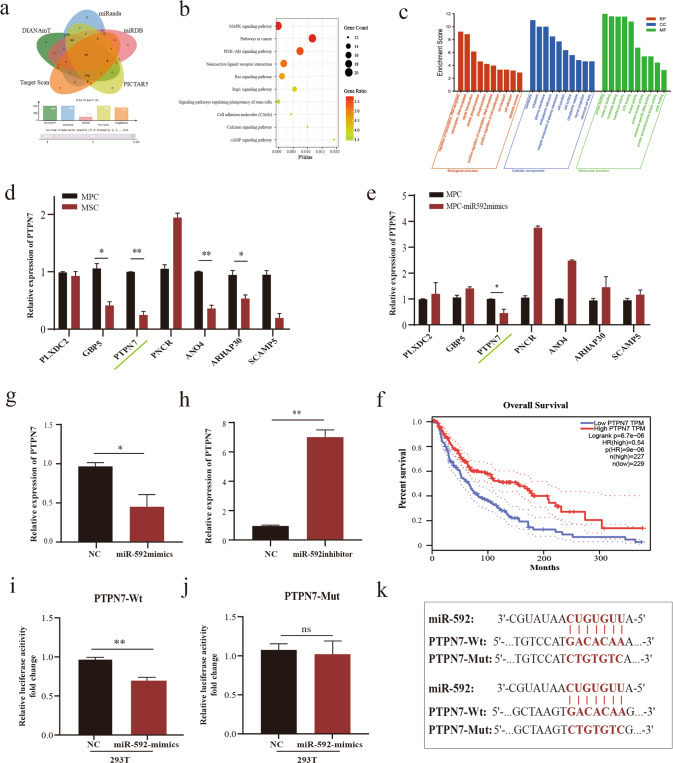
PTPN7 is a direct target of miRNA-592. **a** Target genes of miR-592 were predicted by intersection of multiple databases, *p* < 0.05. **b**, **c** KEGG and GO enrichment analysis were performed for the predicted target genes of miR-592 (DAVID). **d** Target genes of miR-592 expression were verified in MPCs and MSCs. **e** Target genes of miR-592 expression were verified in miR592-OE-MPCs and NC-MPCs. **f** Higher PTPN7 expression is significantly associated with better survival (TCGA database). **g**, **h** PTPN7 expression was regulated by miR-592. **i**–**k** luciferase assay confirmed that PTPN7 was the direct gene target of miR592 (ns not significant; *P < 0.05; **P < 0.01; ***P < 0.001).

miRNA can degrade mRNA and inhibit its protein translation by specifically binding to its target mRNA [24]. Therefore, there is an inverse relationship between a miRNA expression and its gene targets. We conducted a set of bioinformatics analyses to predict gene targets of miR-592. We firstly used database Target Scan and miRWalk to forecast all theoretical miR-592 target genes (Fig. 4a). We then took the intersection of all the predicted genes from the two databases (miRwalk, *n* > 5, *p* < 0.05). Subsequently, we conducted a functional analysis of the predicted target genes of miR-592 via bioinformatics approach. KEGG and GO analysis of all predicted target genes were performed by DAVID (Bioinformatics Resources, DAVID Functional Annotation Bioinformatics Microarray Analysis (ncifcrf.gov). The analysis prioritized MAPK signaling pathway for mechanistic characterization (Fig. 4b, c). Finally, we selected Protein Tyrosine Phosphatase Non-receptor type7, an inhibitor of the MAPK/ERK, for further validation based on: (1) a predicted gene target of miR-592 (Fig. 4a), (2) differential expression (Fig. 4d, e; Supplementary Fig. 3i), (3) association with MAPK/ERK pathway (Fig. 4b), and (4) clinical prognosis analysis (Fig. 4f). TCGA database showed that patients with high PTPN7 expression had significantly longer survival (Fig. 4f).

For biological verification, we measured PTPN7 expression changes upon miR-592 overexpression (miR-592 mimics) or downregulation (miR-592 inhibitor). We found that the expression of PTPN7 was inversely related to the expression of miR-592. That is expected of miRNA-gene target regulation (Fig. 4g, h). Dual-luciferase assay confirmed that PTPN7 is a direct gene target of miR-592 (Fig. 4i–k).

PTPN7 is a member of the protein tyrosine phosphatase (PTP) family. PTP is a signaling molecule that regulates a variety of processes including cell growth and differentiation. The non-catalytic N-terminal of PTPN7 interacts with MAP kinase to specifically inhibit ERK activity [25, 26]. Based on the above dry-lab predictions and experimental validation, we hypothesized that PTPN7 mediates the pro-metastatic activity of EVs miR-592 through MAPK/ERK signaling pathway.

**Fig. 5:**
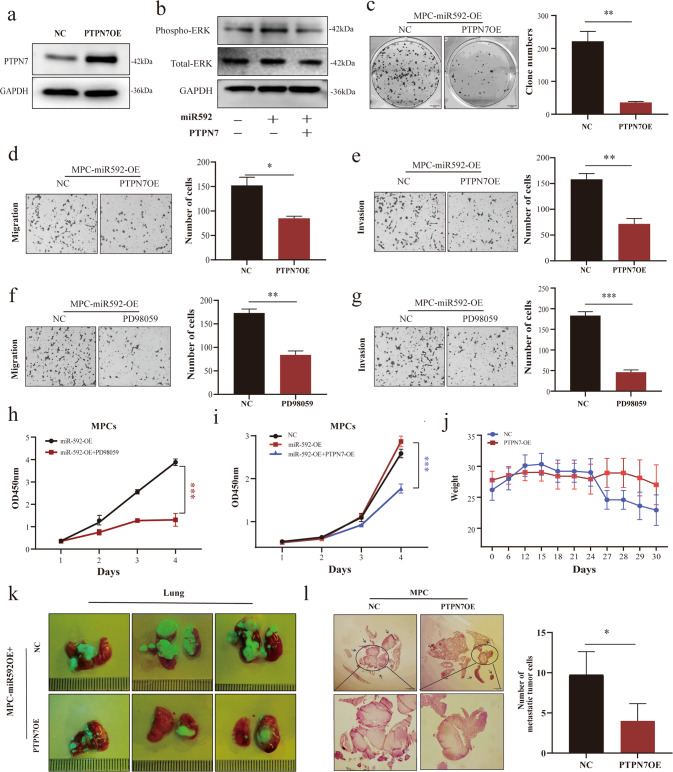
PTPN7 overexpression revered the pro-metastatic activity of miR-592 in MCP cells. **a** The overexpression efficiency of PTPN7 was verified by WB. **b** Western blot analysis on the effect of miR-592 and PTPN7 on phospho-ERK and total-ERK. **c**–**e** Colony formation and Transwell assays revealed that miR-592 mediated oncogenic effects could be attenuated by PTPN7. **f**–**h** Migration, invasion and CCK-8 assays indicated that the oncogenic effect of mir-592 could be mediated by MAPK/ERK inhibitor PD98059 (20 μM). **i** The effect of PTPN7 overexpression on the proliferation of miR-592-OE-MPC cells was determined by the CCK-8 assay. **j** Weight changes in mice injected with control and PTPN7 overexpressed MPC cells. **k** Pulmonary metastasis in mice. The overexpressed PTPN7 was labeled with GFP (green fluorescence). **l** Lung metastasis was observed by H&E staining and quantified by Image J (ns not significant; *P < 0.05; **P < 0.01; ***P < 0.001).

To test this hypothesis, we used PTPN7-overexpressing lentivirus and a specific MAPK/ERK inhibitor PD98059 and conducted a series of rescue experiments. PTPN7 overexpression efficiency in MPC cells was confirmed by WB and PCR (Fig. 5a; Supplementary Fig. 2f, uncropped WB). Western blot analysis of MAPK/ERK activation status showed that ERK phosphorylated protein expression was increased in MPCs upon miR-592 overexpression. PTPN7 overexpression in miR-592-OE-MPCs successfully prevented the miR-592-induced enhancement of MAPK/ERK activation (Fig. 5b; Uncropped WB), we observed the same results in A375, another human malignant melanoma cell line (Supplementary Fig. 3k). Rescue experiments showed that PTPN7 overexpression in MPCs-miR592-OE largely reversed the stimulative effect of miR-592 overexpression on migration, invasion, proliferation, and colony formation in vitro (Fig. 5c–e, i). Subsequently, we conducted functional assays to determine whether MAPK/ERK pathway is required for the pro-metastatic activity of miR-592. For this set of experiments, miR-592-OE-MPCs were treated with the MAPK/ERK specific inhibitor (PD98059, 20 μM, 24 h) prior to functional assessment. We found that PD98059 treatment significantly reduced the enhancement of migration, invasion and colony formation seen upon miR-592 overexpression (Fig. 5f–h; Supplementary Fig. 3j).

To assess the role of PTPN7 in mediating the pro-metastatic activity of miR-592 in vivo, we performed the rescue experiment by injecting through the tail vein in vitro (Methods Animal experiments). At the time of sacrifice, autopsy was performed. From fluorescence photography and H&E staining, it is obvious that overexpression of PTPN7 in miR592-OE-MPCs successfully abrogated the stimulative effect of miR-592 overexpression on MPC metastatic colonization in vivo (Fig. 5j–l; Supplementary Fig. 4b; Supplementary Fig. 3b).

In summary, MSCs can deliver EVs miR-592 to the targeted MPCs. Upon entering MPCs, miR-592 releases the inhibitory effect of PTPN7 on MAPK/ERK signaling pathway by inhibition of its expression which in turn leads to augmentation of metastatic colonization of MPC. Thus, via the EV-miR-592/PTPN7/MAPK axis, melanoma-CSCs can transfer their “metastatic ability” to the low-metastatic non-CSC melanoma cells.

## Discussion

Distant metastasis is the main reason for the high mortality and poor prognosis of cancer patients, including metastatic melanoma [27]. The involvement of EVs in the establishment of metastatic microenvironment has been increasingly appreciated. The intercellular crosstalk between the donor cells and the recipient cells involves the transfer of “biologically active material” including miRNA, mRNA, lncRNA, and proteins. Thus, the biological properties of the recipient cells are submitted to the regulation of EVs interactions [12, 28]. Tumor-derived EVs participate in the formation of metastatic niche [22, 29]. Few studies have shown the EVs crosstalks between the cancer cell and stromal cells or cancer stem cells, as well as the importance of such interaction in cancer progression [30–32]. We recently show that high-metastatic melanoma cells can promote metastasis of low-metastatic melanoma with EVs miR-199a-5p [16].

CSCs are cells with self-renewal capability and play essential roles in oncogenesis and metastatic progression [33–35]. CSCs derived EVs can modify the oncogenic properties of the recipient tumor cells [36]. EVs-miRNAs of CSCs can enhance metastatic progression [37–39]. However, the involvement of EVs secreted by CSCs in melanoma metastasis has not been investigated. Our present study has made the following novel findings that have elucidated a new mechanism of melanoma metastasis based on CSC extracellular vesicles:

### First, EVs from melanoma stem cells can enhance the metastatic colonization capability of melanoma cells

The acquisition of genetic alterations, clonal evolution, and the interactions between tumor cells and stromal cells in the tumor microenvironment (TME) collectively promote cancer progression, metastasis and the development of therapeutic resistance [40, 41]. CSCs can alter TME by directly releasing a variety of bioactive substances [42, 43]. Non-coding RNAs, such as miRNAs in the EVs can alter the TME and mediate cellular communications both locally and distantly [44, 45]. Previous studies on the involvement of EVs in metastasis have focused mainly on the effects of EVs of tumor cells on stromal cells [46]. While CSCs can affect the occurrence and development of cancer, the underlying mechanisms have not been fully confirmed [47, 48]. To our knowledge, at the stage of metastatic colonization, a rate-limiting stage for metastasis, whether extravasated CSCs can modify the metastatic properties of the neighboring non-CSCs and if so, the underlying mechanisms have not been demonstrated prior to this study. We show for the first time that EVs secreted by melanoma-CSCs can enhance the metastasis of non-CSC melanoma cells (Fig. 1).

### Second, EVs miR-592 from highly metastatic melanoma stem cells enhance the metastatic colonization ability of low-metastatic melanoma cells

Prior to this study, the oncogenic activity of miR-592 has only been reported in prostate cancer [19] and thyroid cancer [20]. However, in-depth mechanistic characterization of miR-592 in metastasis is lacking. Its involvement in melanoma is unknown. Further, the function of miR-592 in the context of intercellular crosstalk has not been demonstrated. In this study, we found that miR-592 can be encapsulated by EVs and transported to MPCs, which results in increased metastatic capacity of MPC cells in vitro and in vivo. CSC derived EVs contain stem-specific proteins and regulatory miRNAs that can promote tumor cell metastasis. For example, miR-210-3p from lung CSCs is involved in lung cancer progression by targeting FGFRL1 [49].

### Third, the pro-metastatic activity of CSC EV miRNA-592 is achieved through direct targeting of PTPN7 which in turn activates the MAPK/ERK signaling pathway

In order to explore the mechanisms underlying the pro-metastatic activity of CSCs EVs miR-592, we employed bioinformatics analysis to predict functional and clinically significant gene targets of miR-592 and to enrich related signaling pathways and biological processes. This approach allowed us to prioritize PTPN7, a prognostic predictor of melanoma, as the gene target of miR-592 for mechanistic characterization. We confirmed that PTPN7 is a gene target of miR-592. PTPN7 protein–protein interaction (PPI) network analysis shows that PTPN7 is significantly associated with signaling molecules of the MAPK/ERK pathway. ERK1/2, an extracellular signal-regulated kinases represent the foremost mitogenic pathway in mammalian cells. Phosphorylation activation of ERK1/2 was known to be mediated by MAPK/ERK kinase (MEK) [50–52]. Since the non-catalytic N-terminus of PTPN7 can interact with MAP kinases and suppress the MAP kinase activities, inhibition of PTPN7 by miR-592 targeting will release the inhibitory effect of PTPN7 on MAPK/ERK, leading to its activation.

In conclusion, our study has elucidated that the EVs-mediated crosstalk between the melanoma-CSCs and the non-CSCs melanoma cells is a novel mechanism for melanoma metastasis. We characterized a relatively novel miR-592, a potential prognostic marker for melanoma, is capable of mediating such intercellular crosstalk. Via the EV-miR-592/PTPN7/MAPK axis, melanoma-CSCs can transfer their metastatic ability to the low-metastatic non-CSC melanoma parental cells.

## Supplementary information

Supplementary material

Original Data File

## Data Availability

The data supporting the findings of this study are available within the article and in the supplementary materials.
