# Supplementary material for: Extracellular vesicles microRNA-592 of melanoma stem cells promotes metastasis through activation of MAPK/ERK signaling pathway by targeting PTPN7 in non-stemness melanoma cells
Source: Cell Death Discov. 2022 Oct 27;8:428. doi: 10.1038/s41420-022-01221-z (PMC9614017; doi:10.1038/s41420-022-01221-z)
Supplement: Supplementary file 1 — Supplementary material [file 41420_2022_1221_MOESM1_ESM.docx]

**Supplemental Information:**

# Extracellular vesicles microRNA-592 of melanoma stem cells promotes metastasis through activation of MAPK/ERK signaling pathway by targeting PTPN7 in non-stemness melanoma cells.

Yuhan Zhang^12^, Yan Chen^12^, Lei Shi^12^, Jie Li^3^, Wenjuan Wan^4^ Bowen Li^12^, Xiaoshuang Li^12^, Yuting Chen^12^, Meng Xiang^12^, , Doudou Liu^12^ Hao Chen^12^, Bin Zeng^3^, H. Rosie Xing^12*^ and Jianyu Wang^3*^

^1^State Key Laboratory of Ultrasound in Medicine and Engineering, College of Biomedical Engineering, Chongqing Medical University, Chongqing, 400016, China.

^2^Chongqing Key Laboratory of Biomedical Engineering, Chongqing Medical University, Chongqing 400016, China.

^3^Institute of Life Sciences, Chongqing Medical University, Chongqing, China

^4^Department of Ophthalmology, The First Affiliated Hospital of Chongqing Medical University, Chongqing Key Laboratory of Ophthalmology and Chongqing Eye Institute, Chongqing, China

***Corresponding:**

H. Rosie Xing: [102643@cqmu.edu.cn](mailto:102643@cqmu.edu.cn).

Jianyu Wang: 102758@cqmu.edu.cn.

Tel.: +86-023-63662443 (J.W.), +86-023-63738563 (H.R.X.);

#### Supplementary Tables:

**Table1:** The primers and siRNA sequences:

| GAPDH | Forward: TGATGGGTGTGAACCACGAG |
| --- | --- |
|  | Reverse: AGTGATGGCATGGACTGTGG |
| TBP | Forward: AAGAGAGCCACGGACAACTG |
|  | Reverse: TTCACATCACAGCTCCCCAC |
| β-actin | Forward: CTACCTCATGAAGATCCTGACC |
|  | Reverse: CACAGCTTCTCTTTGATGTCAC |
| U6 | Forward: CTCGCTTCGGCAGCACA |
|  | Reverse: AACGCTTCACGAATTTGCGT |
| PTPN7 | Forward: ACTCCGGGCCTATAGTGGTT |
|  | Reverse: AGTCGAAGTTGGCACACGAT |
| miR-592 | CGCGTTGTGTCAATATGCGATGATGG |
| miR592-mimcs(cy3) | AUUGUGUCAAUAUGCGAUGAUGU |
|  | AUCAUCGCAUAUUGACACAAUUU |
| miR592-NC | UUCUCCGAACGUGUCACGUTT |
|  | ACGUGACACGUUCGGAGAATT |
| miR592-inhibitor | ACAUCAUCGCAUAUUGACACAA |

**Table 2:** Antibodies

| Rab27a | Cat No. 17817-1-AP | 1:2000 | Proteintech |
| --- | --- | --- | --- |
| CD63 | Cat No. 25682-1-AP | 1:1000 | Proteintech |
| CD81 | Cat No. 66866-1-Ig | 1:500 | Proteintech |
| Alix | Cat No. 12422-1-AP | 1:1500 | Proteintech |
| PTPN7 | Cat No. 15286-1-AP | 1:1500 | Proteintech |
| ERK1/2 | Cat No. 11257-1-AP | 1:1000 | Proteintech |
| Phospho-ERK1/2 | Cat No. 28733-1-AP | 1:1000 | Proteintech |
| GAPDH | Cat No. 60004-1-Ig | 1:5000 | Proteintech |
| β-Tubulin | Cat No. 10094-1-AP | 1:2000 | Proteintech |

**.**

**Table 3:** sequence of plasmid:

pGL3-Basic：
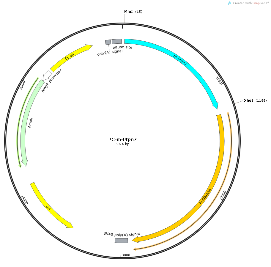


sequence of plasmid:

| **PTPN7- Wt** | ctgaacttacagataggagctggtccttgcaatggattgagagagcagctggagtgctggcttatttaggtgctttcggtgagctcccttccttgtccatgacacaaagagtcagtcaaacagaatgttcctgaaagccattccagatgtagccctgctgatgcggctgcagagatggtgattgcaagatcctcttccattaaggcctttcccacacacaggccagatgcaacagcagggaaaatcctggtctgaccttggtcaaatcttctctatcctacatagactgctcctccccatacacccgtgagtgctggaggaccggctcagaagagaataagggccttgattggcctgcctccatacatatcataaaaggagatgcagaggtaaccagctctagccgttgggatcactgtatgagcccagaagtgatgatgtacctgtcatcctagcactcaggagtagaagcaggtgggttgctgcgagtttgaggtcagctagatctacattttgagtaccaggctagccaggacaacatagcaagattttttttaaggcaatcttgagctctctgctctctggaggagatgagggttgcctctgcttctatggcttccttcctgtcatctgctcccaaccactctctgggggaaaacccagcactctctgctttgcttctgtgacttcagagcatcctttgggtacccatgaagtatcaaagaaagaggagagaagagacaaagagctatgtctgcttcaagaaaacctacctgccgtacctcccctggcttgtatttctttgcagagtgcttatctggttccagaagcaggctgggtggtcaccactccaagcagcatggggctaagaccttcagccctcatcatcatcacttcacagaatttccagggaactcatgatctatctcagggatgaactatgaatcccagaaagccagacaaaccctgagtctcccttgacccggattgggctgcctgtgggggggaaggcagaatactattcctactgtctggtcctcagctcctggatgtgctaagtgacacaagtcctccatgaaaaccccaaggagttggaggagtgactgcctccttgcttcctggcatctctctggagaactcgaagtgcttctggacatagtgtcgaaa |
| --- | --- |
| **PTPN7-mut** | CTGAACTTACAGATAGGAGCTGGTCCTTGCAATGGATTGAGAGAGCAGCTGGAGTGCTGGCTTATTTAGGTGCTTTCGGTGAGCTCCCTTCCTTGTCCATctgtgtcAGAGTCAGTCAAACAGAATGTTCCTGAAAGCCATTCCAGATGTAGCCCTGCTGATGCGGCTGCAGAGATGGTGATTGCAAGATCCTCTTCCATTAAGGCCTTTCCCACACACAGGCCAGATGCAACAGCAGGGAAAATCCTGGTCTGACCTTGGTCAAATCTTCTCTATCCTACATAGACTGCTCCTCCCCATACACCCGTGAGTGCTGGAGGACCGGCTCAGAAGAGAATAAGGGCCTTGATTGGCCTGCCTCCATACATATCATAAAAGGAGATGCAGAGGTAACCAGCTCTAGCCGTTGGGATCACTGTATGAGCCCAGAAGTGATGATGTACCTGTCATCCTAGCACTCAGGAGTAGAAGCAGGTGGGTTGCTGCGAGTTTGAGGTCAGCTAGATCTACATTTTGAGTACCAGGCTAGCCAGGACAACATAGCAAGATTTTTTTTAAGGCAATCTTGAGCTCTCTGCTCTCTGGAGGAGATGAGGGTTGCCTCTGCTTCTATGGCTTCCTTCCTGTCATCTGCTCCCAACCACTCTCTGGGGGAAAACCCAGCACTCTCTGCTTTGCTTCTGTGACTTCAGAGCATCCTTTGGGTACCCATGAAGTATCAAAGAAAGAGGAGAGAAGAGACAAAGAGCTATGTCTGCTTCAAGAAAACCTACCTGCCGTACCTCCCCTGGCTTGTATTTCTTTGCAGAGTGCTTATCTGGTTCCAGAAGCAGGCTGGGTGGTCACCACTCCAAGCAGCATGGGGCTAAGACCTTCAGCCCTCATCATCATCACTTCACAGAATTTCCAGGGAACTCATGATCTATCTCAGGGATGAACTATGAATCCCAGAAAGCCAGACAAACCCTGAGTCTCCCTTGACCCGGATTGGGCTGCCTGTGGGGGGGAAGGCAGAATACTATTCCTACTGTCTGGTCCTCAGCTCCTGGATGTGCTAAGTctgtgtcGTCCTCCATGAAAACCCCAAGGAGTTGGAGGAGTGACTGCCTCCTTGCTTCCTGGCATCTCTCTGGAGAACTCGAAGTGCTTCTGGACATAGTGTCGAAA |

**Table 4:** Tumor type abbreviations:

| BLCA | Bladder Urothelial Carcinoma |
| --- | --- |
| BRCA | Breast invasive carcinoma |
| CHOL | Cholangio carcinoma |
| COAD | Colon adenocarcinoma |
| ESCA | Esophageal carcinoma |
| HNSC | Head and Neck squamous cell carcinoma |
| KICH | Kidney Chromophobe |
| KIRC | Kidney renal clear cell carcinoma |
| KIRP | Kidney renal papillary cell carcinoma |
| LIHC | Liver hepatocellular carcinoma |
| LUAD | Lung adenocarcinoma |
| LUSC | Lung squamous cell carcinoma |
| PRAD | Prostate adenocarcinoma |
| STAD | Stomach adenocarcinoma |
| UCEC | Uterine Corpus Endometrial Carcinoma |

#### 2. Supplementary Methods:

**2.1. miRNA sequencing:**

BGI-shenzhen Carry out specific RNA extraction and miRNA sequencing services. The specific sequencing method is as follows: library was prepared with 1 μg total RNA for each sample. Total RNA was purified by electrophoretic separation on a 15% urea denaturing polyacrylamide gel electrophoresis (PAGE) gel and small RNA regions corresponding to the 18–30 nt bands in the marker lane (14-30 ssRNA Ladder Marker, TAKARA) were excised and recovered. Then the 18–30 nt small RNAs were ligated to adenylated 3’ adapters annealed to unique molecular identifiers (UMI), followed by the ligation of 5’adapters. The adapter-ligated small RNAs were subsequently transcribed into cDNA by SuperScript II Reverse Transcriptase (Invitrogen, USA) and then several rounds of PCR amplification with PCR Primer Cocktail and PCR Mix were performed to enrich the cDNA fragments. The PCR products were selected by agarose gel electrophoresis with target fragments 110~130 bp, and then purified by QIAquick Gel Extraction Kit (QIAGEN, Valencia, CA). The library was quality and quantitated in two methods: check the distribution of the fragments size using the Agilent 2100 bioanalyzer, and quantify the library using real-time quantitative PCR (QPCR) (TaqMan Probe). The final ligation PCR products were sequenced using the BGISEQ-500 platform (BGI-Shenzhen, China).

#### Supplementary figures and legends:


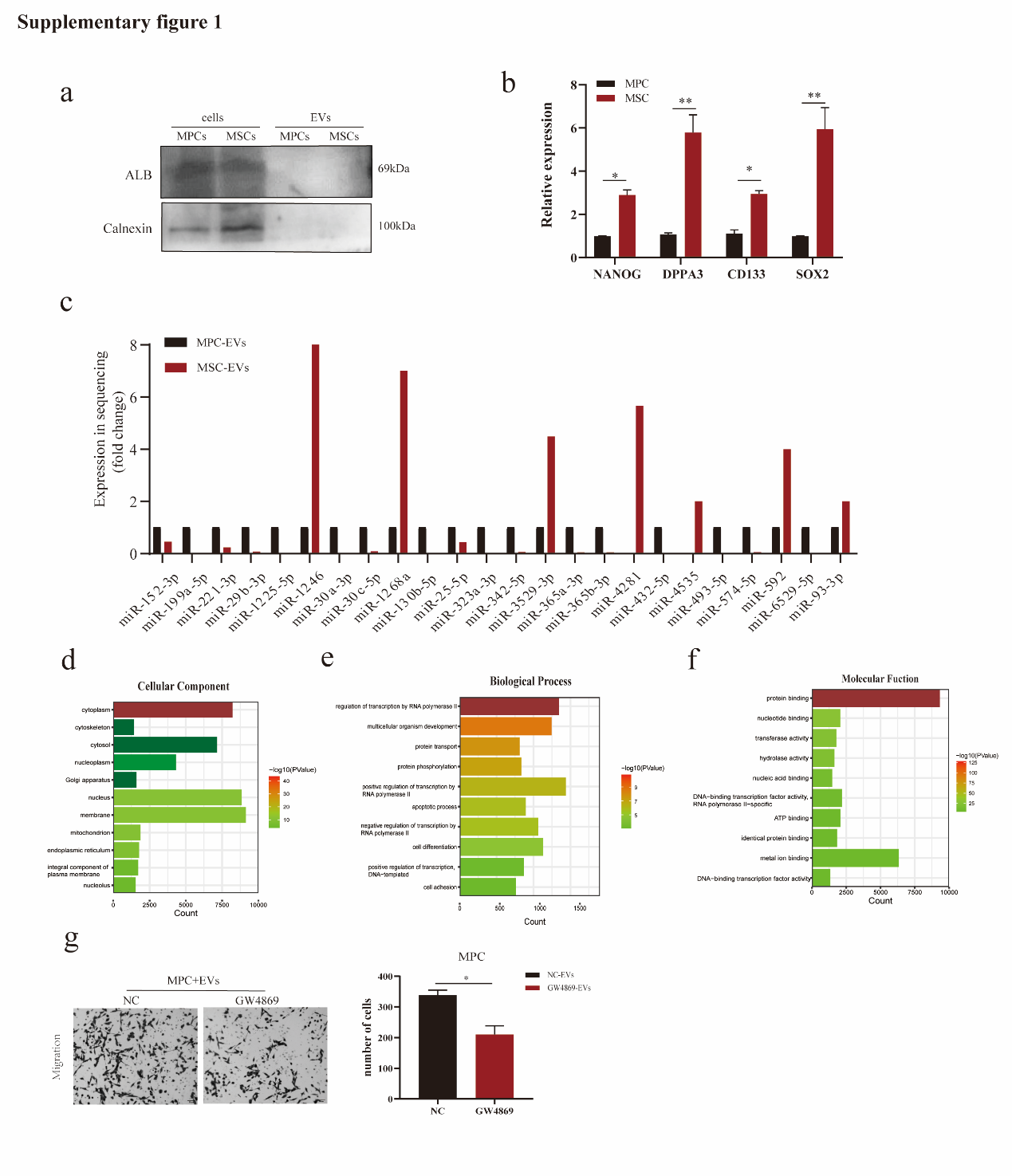


1. Small EVs negative markers by Western Blot. (b) The expression of stemness markers

were significantly increased in MSC cells. (c) Differential miRNAs in small EVs sequencing, the bar charts and statistics were produced by GraphPad Prism 8.0.2. (d-f) GO enrichment analysis of downstream target genes of miR-592; analytical data from the website DAVID and the visualization is presented in R language 4.0.4. (g) GW4869 inhibits EVs secretion and attenuates MPCs metastatic ability.


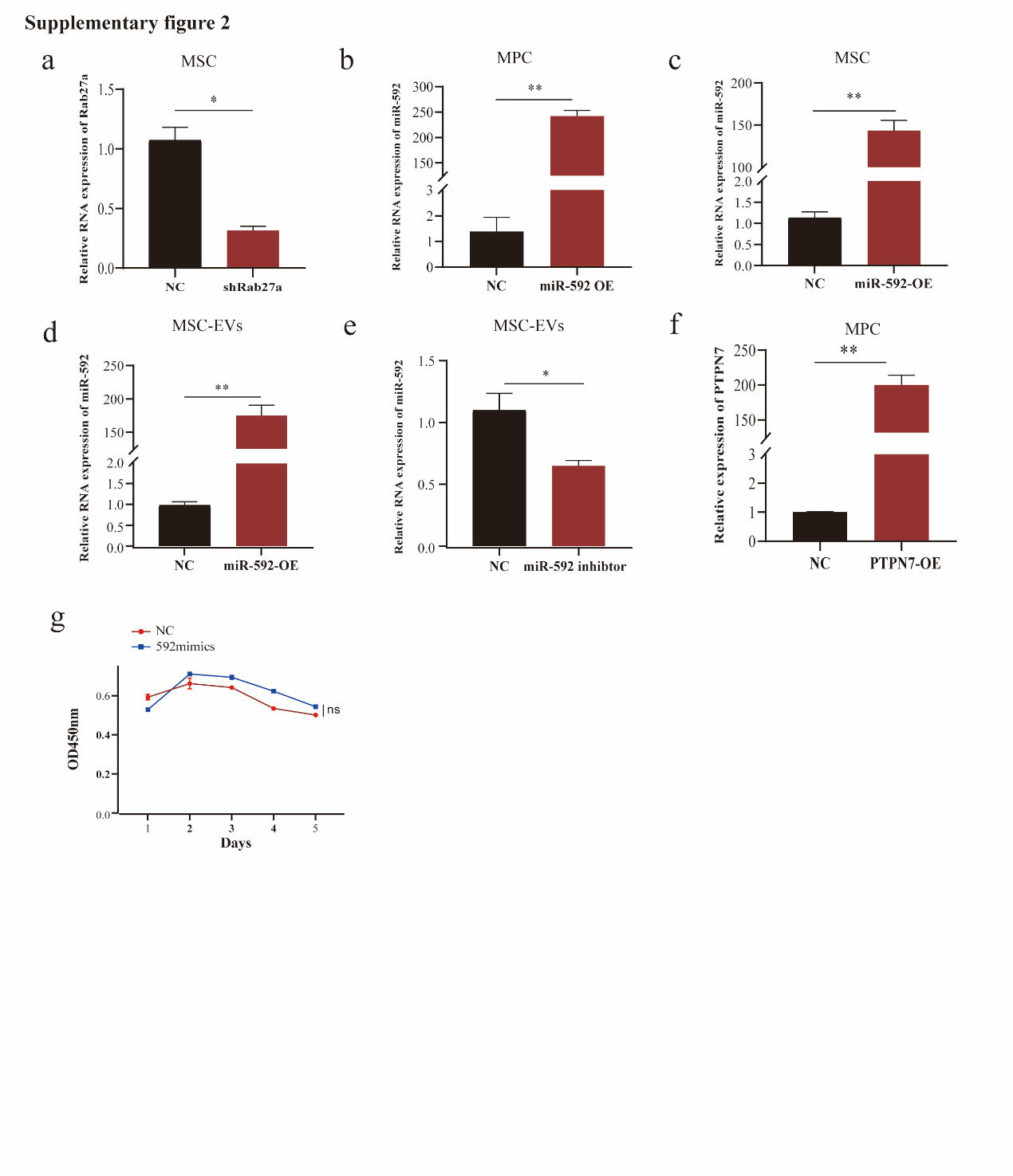


(a-c) q-PCR verification of the efficiency of miR-592 mimics and inhibitor in MPCs and MSCs. (d-e) q-PCR verification of the efficiency of miR-592mimics and inhibitor in MPC-EVS and MSC-EVS. (f) The CCK-8 assay to determine the effect of miR-592 overexpression via miR-592 mimics on the proliferation of MPCs. (g) Ptpn7 is highly related to the genes of the MAPK family. Data from website STRING, visualization done by Cytoscape3.9.1. (h) The efficiency of Ptpn7 overexpression lentivirus (GenePharma, Shanghai). (i) Differential expression of stemness markers in MPC-miR592-OE cells by q-PCR. (j) Differential expression of stemness markers in MSC-miR592-inhibitor cells by q-PCR.


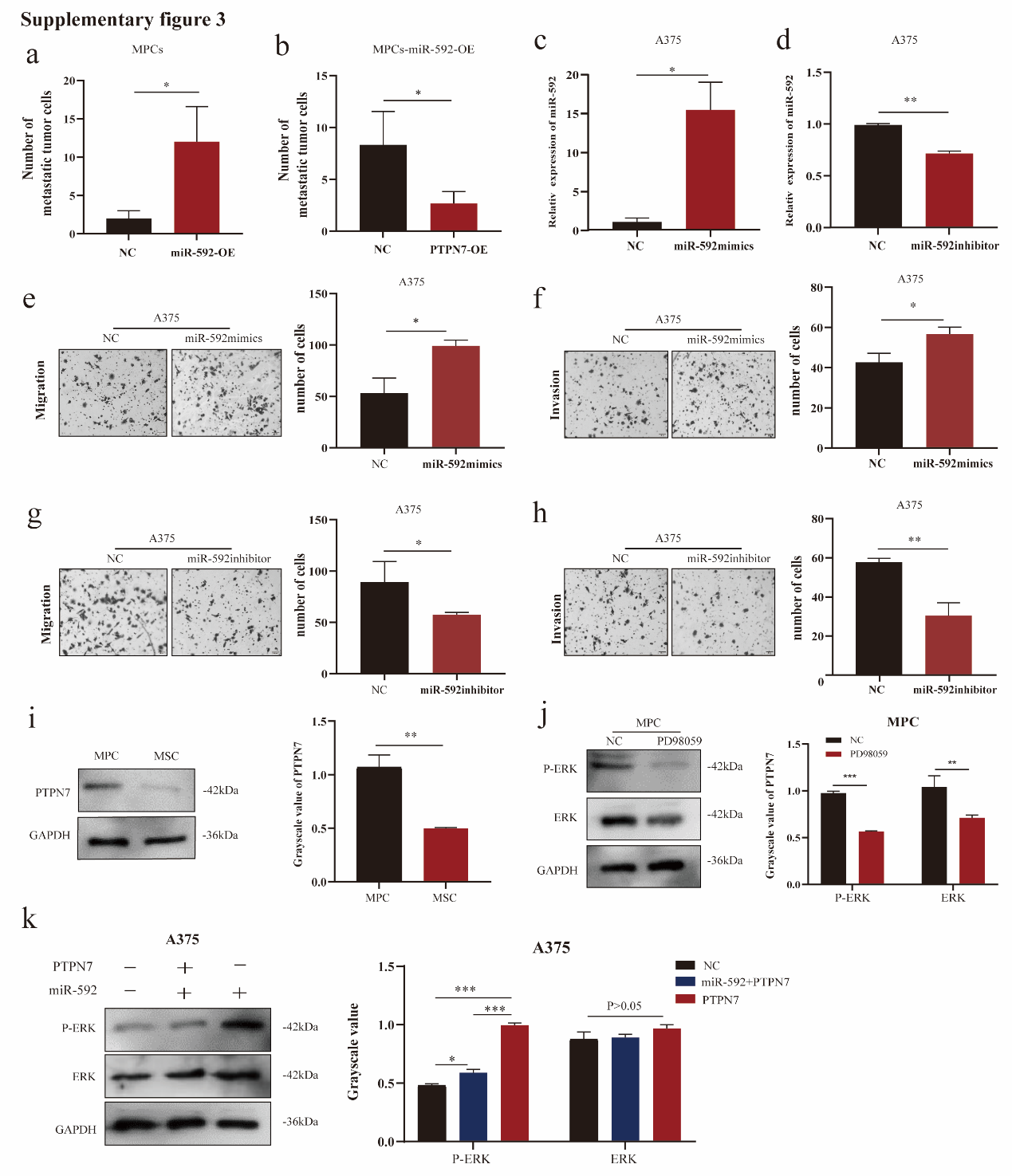
(a)Statistics on the number of metastases in fig.3g. (b) Statistics on the number of metastases in fig.5g. (c-d) q-PCR detect of the efficiency of miR-592 mimics and inhibitor in A375 cells. (e-f). miR-592-mimics promotes migration and invasion of A375 cells. (g-h) miR-592-inhibitor attenuates migration and invasion of A375 cells. (i) Protein levels of PTPN7 were downregulated in MSCs. (j) PD98059 inhibits ERK and phosphorylates ERK protein levels. (k) In A375 cells, miR-592 promoted ERK phosphorylation, while overexpression of PTPN7 could attenuated this effect.


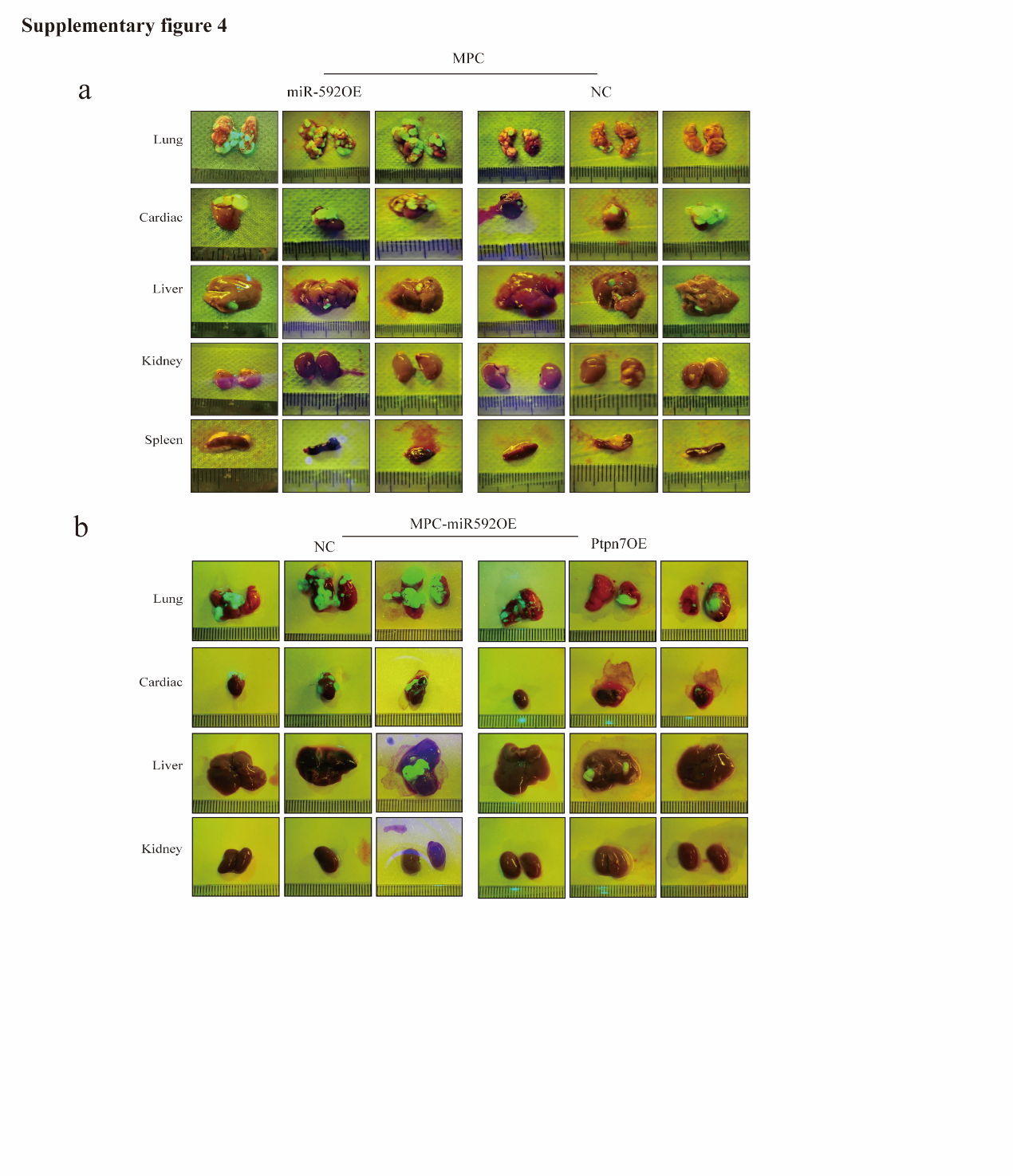


(a) MPC-miR592OE cells were injected through the tail vein of nod-scid mice (n=5). (b) MPC-miR592OE-Ptpn7OE cells were injected through the tail vein of nod-scid mice (n=5). Hearts, livers, spleens, lungs, and kidneys were dissected 30 days after tumor cell inoculation to examine the extend of macrometstases (green).
