# Supplementary figures and images for: Extracellular vesicles microRNA-592 of melanoma stem cells promotes metastasis through activation of MAPK/ERK signaling pathway by targeting PTPN7 in non-stemness melanoma cells
Source: Cell Death Discov. 2022 Oct 27;8:428. doi: 10.1038/s41420-022-01221-z (PMC9614017; doi:10.1038/s41420-022-01221-z)

Uncropped Western blot images


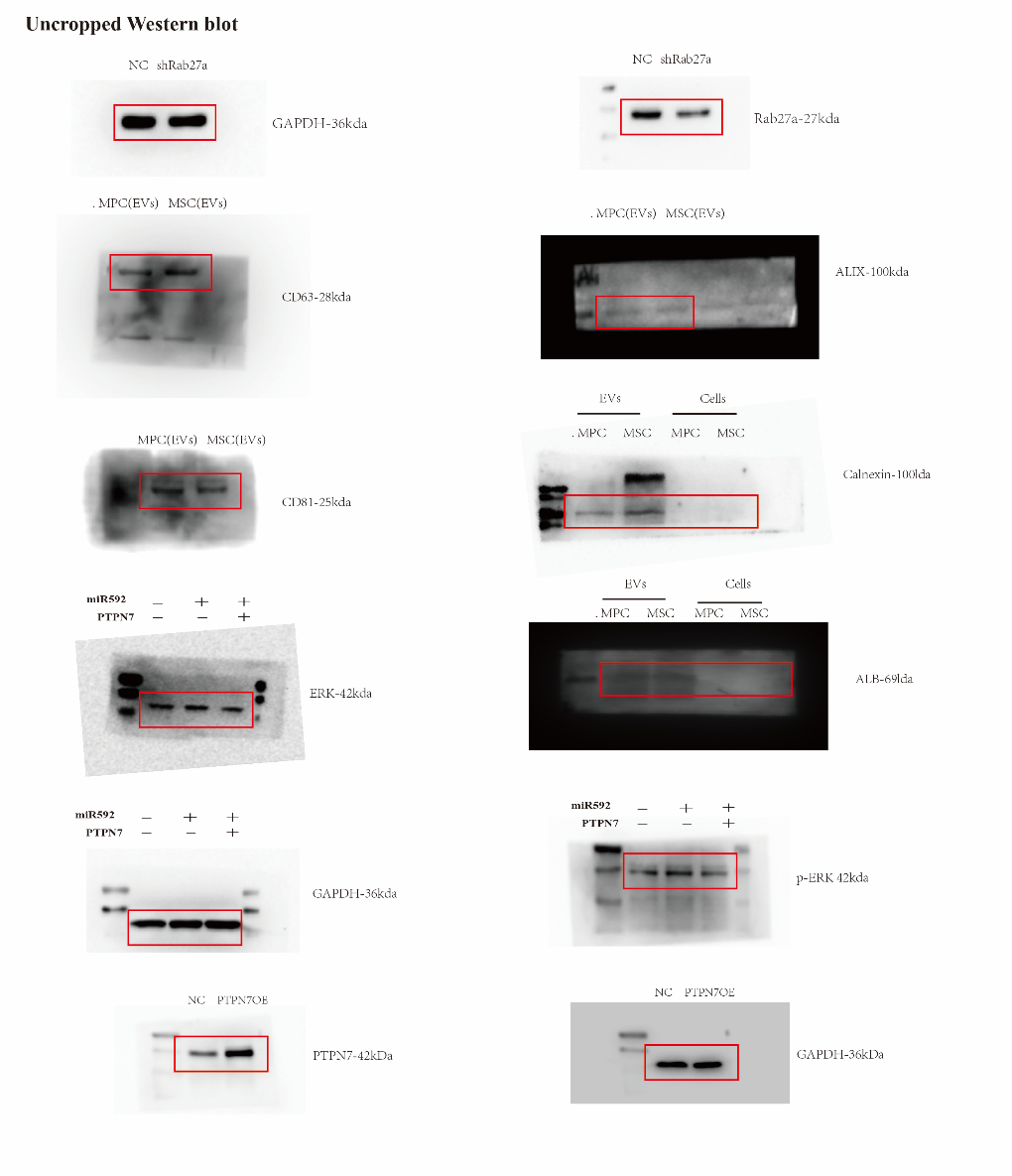


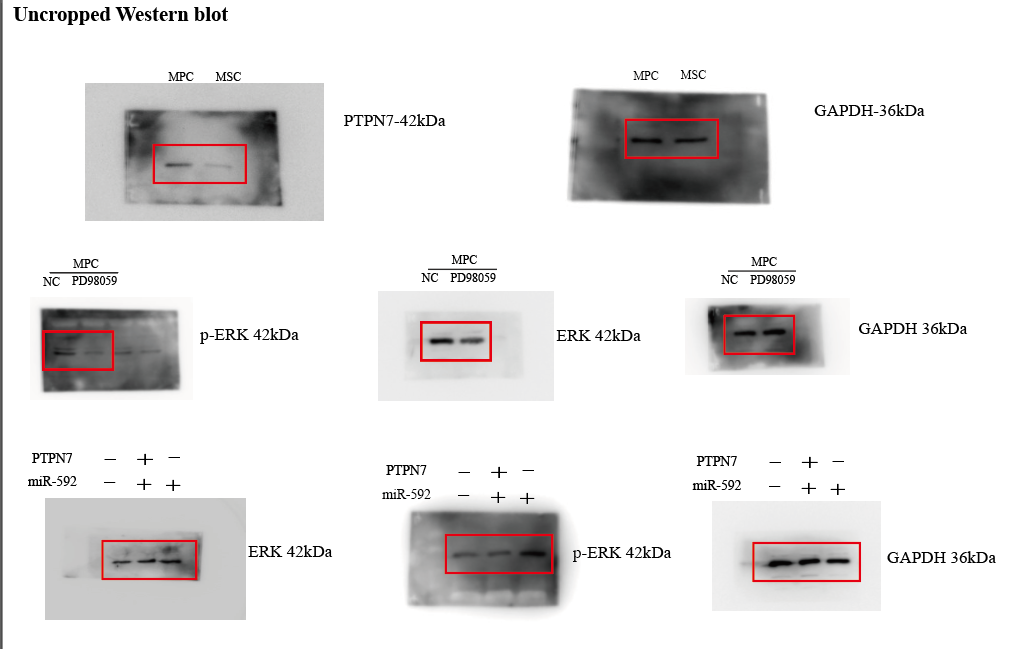

Supplement: Supplementary file 2 — Original Data File [file 41420_2022_1221_MOESM2_ESM.docx]
